# Molecular Docking and QSAR Study of 2-Benzoxazolinone, Quinazoline and Diazocoumarin Derivatives as Anti-HIV-1 Agents

**DOI:** 10.22037/ijpr.2019.1100746

**Published:** 2019

**Authors:** Kamyar Faghihi, Mahdieh Safakish, Tannaz Zebardast, Zahra Hajimahdi, Afshin Zarghi

**Affiliations:** a *Department of Pharmaceutical Chemistry, Faculty of Pharmaceutical Chemistry, Tehran Medical Sciences, Islamic Azad University, Tehran, Iran. *; b *Active Pharmaceutical Ingeredients Research Center, Tehran Medical Sciences, Islamic Azad University, Tehran, Iran.*; c *Department of Medicinal Chemistry, School of Pharmacy, Shahid Beheshti University of Medical Sciences, Tehran, Iran. *; d *Food and Drug Department, Iran University of Medical Sciences, Tehran, Iran.*

**Keywords:** Anti-HIV, Docking, QSAR, Multiple linear regressions, Stepwise

## Abstract

A series of 2-benzoxazolinone, diazocoumarin and quinazoline derivatives have been shown to inhibit HIV replication in cell culture. To understand the pharmacophore properties of selected molecules and design new anti-HIV agents, quantitative structure–activity relationship (QSAR) study was developed using a descriptor selection approach based on the stepwise method. Multiple linear regression method was applied to relate the anti-HIV activities of dataset molecules to the selected descriptors. Obtained QSAR model was statistically significant with correlation coefficient R^2^ of 0.84 and leave one out coefficient Q^2^ of 0.73. The model was validated by test set molecules giving satisfactory prediction value (R^2^_test_) of 0.79. Molecules also were docked on HIV integrase enzyme and showed important interactions with the key residues in enzyme active site. These data might be helpful for design and discovery of novel anti-HIV compounds.

## Introduction

AIDS was first reported in 1981, and subsequently isolated in 1983. More than 70 million people have been infected with and about 35 million people have died of HIV since the beginning of the HIV epidemic. According to WHO reports, 36.7 million people were living with HIV at the end of 2015 (1). After discovery of HIV role in acquired immunodeficiency syndrome (AIDS), all researches have been conducted toward understanding the viral biology and identifying new targets for clinical intercession. The progress in this area has revealed the seven stages of the HIV life cycle including: viral entry, reverse transcription, integration, gene expression, assembly, budding, and maturation (2, 3). Common anti-retroviral drugs based on their inhibitory mechanisms target viral entry, reverse transcription (RT; nucleoside and non-nucleoside inhibitors of the viral reverse transcriptase), integration (IN: integrase inhibitors) and viral maturation (PR: protease inhibitors) (4). The highly active anti-retroviral therapy (HAART) is currently in use as a standard therapeutic perspective. AIDS-related deaths have decreased by 45% since the peak in 2005 by recent advances in anti-HIV drugs and regimens, but still need much more to do (5). Despite of meaningful progresses in HIV therapy, current antiviral chemotherapy still suffers from side effects and revealing drug resistance. Thus, design and discovery of novel therapeutic agents featuring new structures and scaffolds are essential. 

Literature study indicates use of various cheminformatics methods in drug design and discovery. Among these methods, the quantitative structure-activity relationship (QSAR) is a powerful method which established a link between biological activity of drugs and chemical structure or with structural features (6, 7). A good QSAR model describes how biological activity or property of a set of molecules can be differed as a function of molecular descriptors derived from the chemical structure. QSAR methods are low-cost and faster than *in-vitro* and *in-vivo* assays. By QSAR we build validated models by using analysis methods to determine linear or non-linear relationship between the structures and their activities. Using Obtained QSAR models for quantitatively predicting the activities of candidate structures, we avoid extra costs for drug design and discovery like synthesis and bioactivity evaluation (8).

In Recent years we have focused on design and synthesis of various structures as anti-HIV agents. We developed some novel anti-HIV agents featuring 4-oxo-1,4-dihydroquinoline, 4-oxo-4*H*-pyrido [1,2-*a*] pyrimidine, 2-benzoxazolinone, diazocoumarin and quinazoline scaffolds (9-11). In this study, QSAR analysis was carried out on a series of 2-benzoxazolinone, diazocoumarin and quinazoline derivatives to explore a quantitative relationship between their anti-HIV activities and structural properties. Since synthesized compounds were designed based on HIV integrase inhibitors pharmacophores, we also performed a molecular docking study to predict their interaction with HIV integrase. HIV integrase represents one of the key enzymes of virus that catalyzes the insertion of the pro-viral DNA into the genome of infected CD4 cells (12). Obtained results would be helpful in screening new compounds for anti-HIV activity. 

*Methods*

*Data set*

A set of 29 2-benzoxazolinone, diazocoumarin and quinazoline derivatives with their correspondent activity data reported previously from our laboratory were collected to perform QSAR study (13, 14). The biological activity of dataset was given as inhibition rate of p24 expression values. The inhibition rate of p24 expression values were converted to their logarithmic values (Log IR). The Log IR of p24 was used as the dependent variable for the QSAR analysis. The total set of molecules was divided randomly into a training set (24 compounds) for generating QSAR model and a test set (5 compounds) for validation of the model quality. The general chemical structures and inhibition rate of p24 expression values of all of the compounds are listed in Table 1.

*Molecular descriptors and geometry optimizing*

The chemical structures of the molecules were drawn using the HyperChem software (version 7.0; Alberta, Canada). The pre-optimization was conducted using the molecular mechanics force field (MM+) procedure and then low-energy conformers were obtained by the semi-empirical method AM1 using the Polak-Ribiere algorithm until the root mean square gradient was 0.01 kcal mol^-1^.

*Data Reduction-Data pretreatment*

The resultant geometries were transferred into the PaDEL and Dragon software packages to calculate the descriptors. PaDEL is software that currently calculates about 1444 1D, 2D and 3D descriptors. The descriptors are calculated using the Chemistry Development Kit such as atom type electro topological state descriptors, Crippen′s log P and MR, extended topo-chemical atom (ETA) descriptors, McGowan volume, molecular linear free energy relation descriptors, ring counts, count of chemical substructures identified by Laggner, and binary fingerprints and count of chemical substructures identified by Klekota and Roth (15).

Dragon is software that calculates molecular descriptors that are divided into 30 logical blocks (Table 2). the simplest atom types, functional groups and fragment counts, topological and geometrical descriptors, three-dimensional descriptors, and several properties estimation (such as log P), drug-like and lead-like alerts (such as the Lipinski′s alert), 2D autocorrelations, charge descriptors, aromaticity indices, geometrical descriptors, WHIM descriptors, GETAWAY descriptors and empirical descriptors are some examples of these descriptors (16, 17).

After merging resulted data obtained from two software packages, totally 2942 descriptors were calculated and then analyzed by calculation of correlations among descriptors and with the activity of the molecules for redundancy. After identification Collinear descriptors using correlation coefficient cut-off value of 0.9, those that contain a high percentage (>90%) of identical values for all the 29 molecules were discarded. For any given pair of descriptors exhibiting a correlation coefficient value exceeding 0.9, the one exhibiting the highest correlation with the activity was remained and the rest were subjected to removal. Constant or near constant descriptors (> 90%) for all the 29 molecules were also eliminated. The remaining descriptors were collected in an n×m data matrix (D), where n = 29 and m = 379 are the numbers of the compounds and the descriptors, respectively.

**Table 1:** Experimental and predicted LOG IR of dataset molecules by SW–MLR model

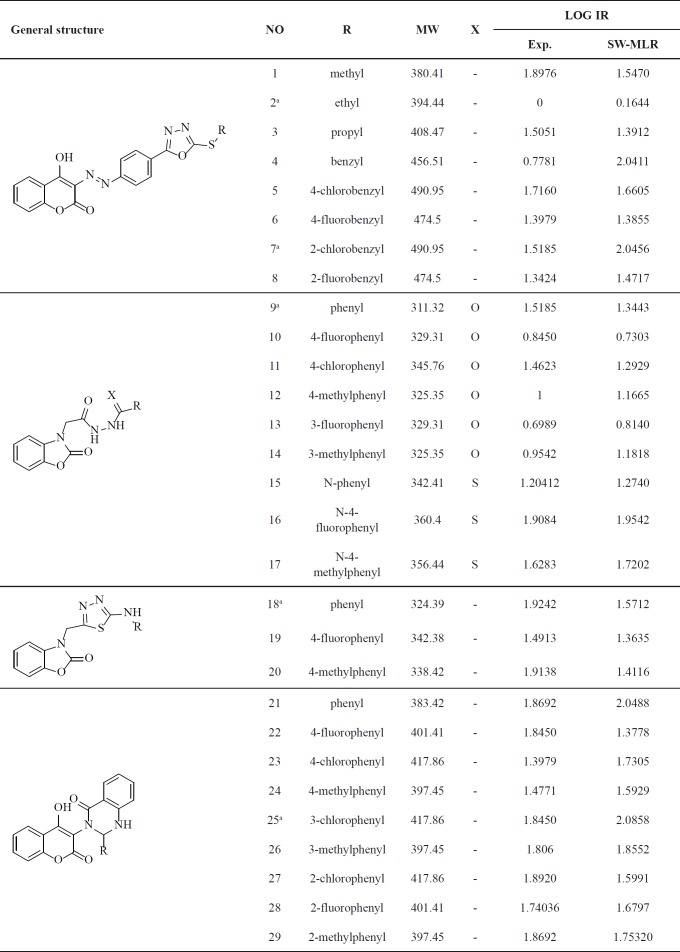

^a^ test set

**Table 2:** 30 logical blocks of Dragon software

**1**	Constitutional	**16**	RDF descriptors
**2**	Ring descriptors	**17**	3D-MoRSE descriptors
**3**	Topological indices	**18**	WHIM descriptors
**4**	Walk and path counts	**19**	GETAWAY descriptors
**5**	Connectivity indices	**20**	Randic molecular profiles
**6**	Information indices	**21**	Functional groups count
**7**	2D matrix-based descriptors	**22**	Atom-centered fragments
**8**	2D autocorrelations	**23**	Atom-type E-state indices
**9**	Burden eigen values	**24**	CATS 2D
**10**	P-VSA-like descriptors	**25**	2D Atom Pairs
**11**	ETA indices	**26**	3D Atom Pairs
**12**	Edge adjacency indices	**27**	Charge descriptors
**13**	Geometrical descriptors	**28**	Molecular properties
**14**	3D matrix-based descriptors	**29**	Drug-like indices
**15**	3D autocorrelations	**30**	CATS 3D

**Table 3:** Details of name of the descriptors were used in model construction

**Variable**	**Descriptor type**	**Definition**
R3u+	GETAWAY descriptors	R maximal autocorrelation of lag 3 / unweighted
R3v+	GETAWAY descriptors	R maximal autocorrelation of lag 3 / weighted by van der Waals volume
IDDE	Information indices	mean information content on the distance degree equality
Mor11m	3D-MoRSE descriptors	3D-MoRSE signal-11/weighted by atomic masses

**Table 4:** Statistical results of QSAR model

**QSAR model**	**Training set**					**Test set R** **2**
	**R** **2**	**RMSE**	**Q** **2** **LOO**	**RMSE LOO**	**F(4,19)**	
SW-MLR	0.84	0.20821	0.73	0.72	25.33	0.79

**Table 5:** Correlation coefficient matrix of the selected descriptors by SW-MLR

	**R3u+**	**R3v+**	**IDDE**	**Mor11m**
R3u+	1	0.432	-0.153	0.241
R3v+		1	-0.0102	0.246
IDDE			1	-0.533
Mor11m				1

**Table 6:** R2 and Q2 values of models after several Y-randomization test

**Iteration**	**R** **2**	**Q** **2** **LOO**	**Iteration**	**R** **2**	**Q** **2** **LOO**
1	0.22	-0.30	11	0.23	-0.23
2	0.23	-0.23	12	0.25	-0.30
3	0.24	-0.23	13	0.25	-0.24
4	0.28	-0.13	14	0.24	-0.29
5	0.29	-0.27	15	0.17	-0.36
6	0.21	-0.27	16	0.16	-0.38
7	0.27	-0.31	17	0.19	-0.31
8	0.20	-0.39	18	0.17	-0.34
9	0.21	-0.29	19	0.25	-0.18
10	0.22	-0.26	20	0.32	-0.18

**Figure 1:**
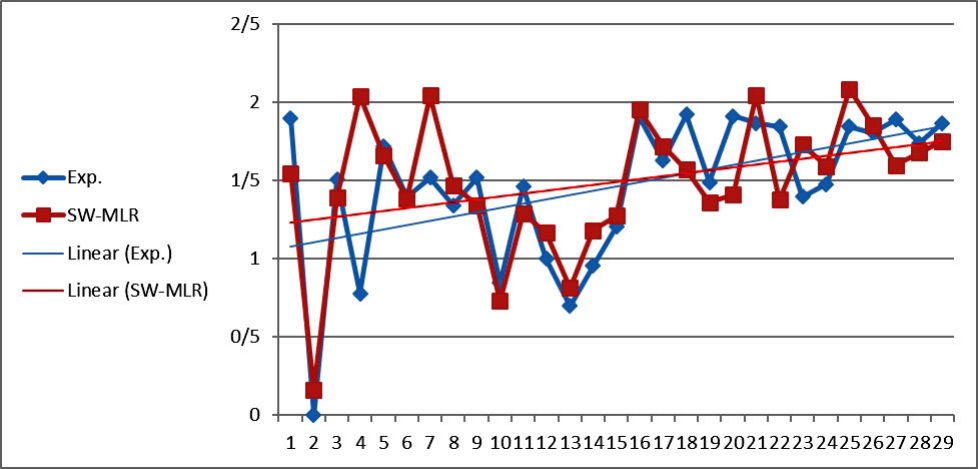
The predicted values of LOG IR using the SW-MLR model versus the experimental values

**Figure 2:**
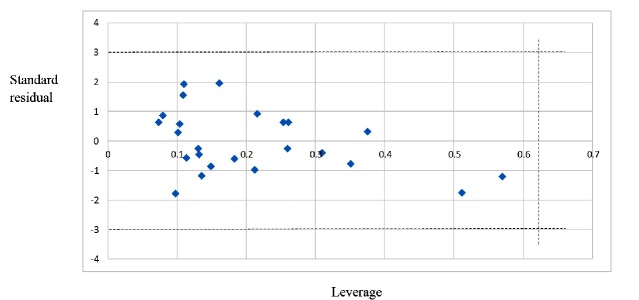
The William plot for the SW-MLR model

**Figure 3:**
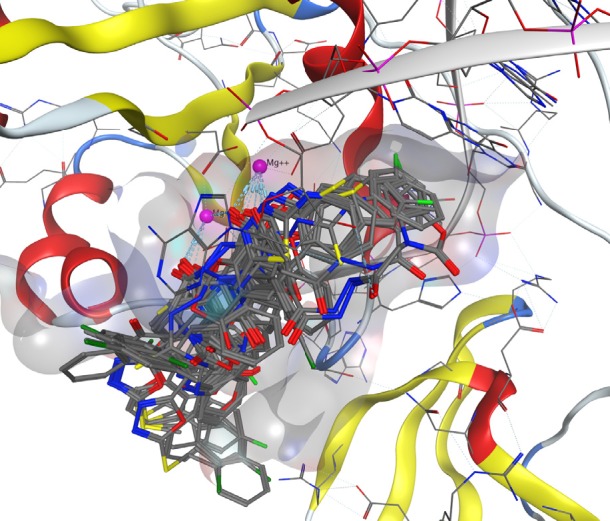
Superimposition of best docked pose of all compounds in HIV integrase active site

**Figure 4:**
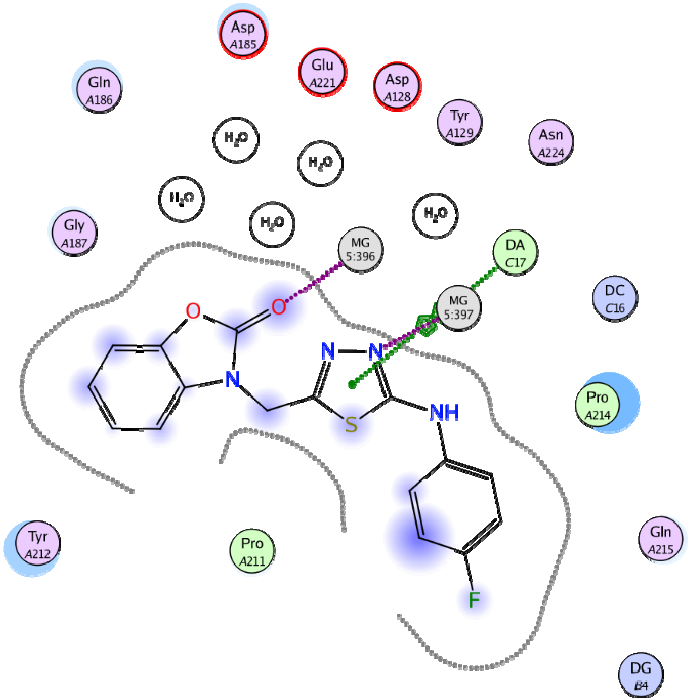
Best docked pose of compound 19 in interaction with HIV integrase residues

**Figure 5:**
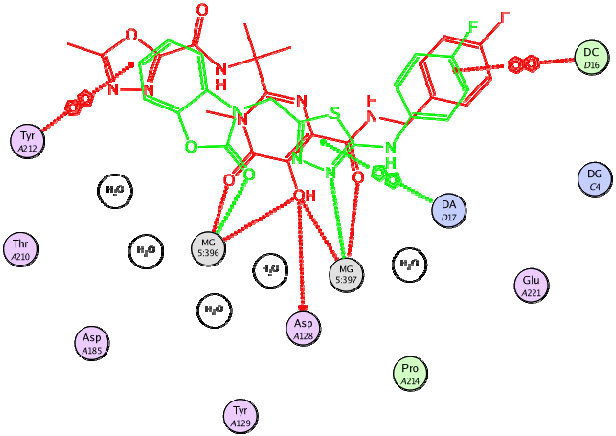
Compound 19 (in green color) superimposed on the co-crystalized ligand (in red color)

**Figure 6:**
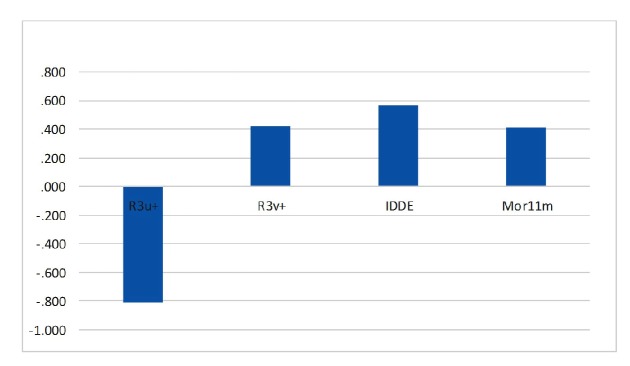
Standardized coefficients versus descriptor values in SW-MLR

*Subjective feature selection-Variable selection techniques*

A major problem of QSAR is the high dimensions of the feature space; therefore, feature selection is the most important step in this study. Variable and subjective feature selection and feature extraction has become the spotlight of much researches in the areas of application for which datasets with tens or hundreds or thousands of variables are available. These areas include pattern recognition, machine learning, statistics and data mining communities, text processing of internet documents, gene expression array analysis, and combinatorial chemistry. The aim of feature selection is to choose a subset of input variables by eliminating features, which are irrelevant or of no predictive information to obtain as much information as possible from a reduced amount of features in order to determining the best subset of variables used in the final QSAR model. The main objective of variable selection is to achieve a balance between simplicity and fit. Feature selection has been proven in both theory and practice to be effective in increasing predictive accuracy and reducing complexity of results. Feature selection in supervised learning has a main goal of finding a feature subset that produces higher classification accuracy (18).

*Stepwise (SW) regression method*

Several feature selection algorithms are available. Each algorithm has its own strength and weakness. Stepwise method is a combination of the forward and backward selection techniques to select the statistically meaningful descriptors by an automatic procedure. Stepwise regression is based on two different strategies, forward selection (FS) and backward elimination (BE). Forward selection begins with no variable presented in the model and testing the addition of each variable improving the model fitness and backward elimination with all variables and testing the removing of candidate variables which can improve the model by being deleted (19). Here in our study we utilized IBM SPSS Statistics V.22 for SW process. 

*Multiple Linear Regressions*

Multiple linear regression (MLR) is the most common form of linear regression analysis.  As a predictive analysis, the multiple linear regressions are used to explain the relationship between one continuous dependent variable from two or more independent variables. In other words, a linear relationship is assumed between the dependent variable and the independent variables. The independent variables can be continuous or categorical (20).

In this research, the available data set was a matrix with size of 24×379 where are total number of training group and variables, respectively. At the end of this stage, the best set of the calculated descriptors was selected by using SW. The SPSS software was employed for the SW-MLR analysis.

*Docking procedure*

Docking study was performed using the Autodock Vina (21) in which the HIV integrase protein was selected from the Protein Data Bank (PDB code: 3OYA). The protein and ligands were prepared in Autodock tools 1.5.6 from MGL Tools package (22). First of all, the co-crystalized ligand and all water molecules were removed from protein crystal. Polar hydrogens were added, non-polar hydrogens were merged, and finally Kallman charge and atom type parameters were added to the protein. A grid box with 20×20×20 dimensions was set to cover active site. All molecules of data set were docked in the active site and the bioactive conformations were generated using Autodock Vina. All ligand-receptor interactions including π-π stacking, π cationic and hydrophobic interactions were detected on the basis of docking results. MOE (Molecular Operating environment) program was used for visualization and analysis of docking results.

## Results

*SW-MLR Model*

After splitting of the data set into the training and test sets, stepwise (SW) was performed to select descriptors correlated with the activity based on the training set samples. Multiple linear regressions analysis with stepwise selection was used to model the structure–activity relationships. The SW-MLR was run and among the resulted models, one model with the highest statistical quality with four most relevant descriptors to build the model was selected. The obtained linear equation for the selected descriptors based on SW-MLR is given as follows:

*LOG IR = -5.79161(±1.3798) -28.80985(±3.67541)R3u+73.15802(±17.94405)R3v+1.59486(±0.30495)IDDE +0.89459(±0.24401)Mor11m*

The model was then used to predict LOG IR values for the compounds in the dataset. The MLR model selected descriptors and their definition are shown in Table 3. The prediction results are given in Table 1. 

The statistical parameters of this model are shown in Table 4. The value of the R^2 ^of SW-MLR model was obtained to be 0.84 for the training set and 0.79 for the test set. The derived model was validated by leave-one-out (LOO) cross-validation process. For LOO cross-validation, a data point is removed from the set, and the model is recalculated. The predicted activity for that point is then compared to its actual value. This is repeated until each data point is left off once. Cross-validation parameters are shown in Table 4. The predicted values of LOG IR for the compounds in training, and test sets using the SW-MLR model have been plotted versus the experimental values of it (Figure 1).

The inter-correlation results between the four selected descriptors in SW-MLR model (Table 5) indicated that the correlation coefficient value of each pair descriptors was less than

 – 0.53; therefore, selected descriptors by stepwise method were completely independent (23).

The model was also validated by applying Y-randomization test. Several random shuffles were performed on dependent variable (anti-HIV activity) and new QSAR models were built. The low R^2^ and Q^2^_LOO_ values show that the good results in obtained models are not because of a chance correlation (Table 6).

After internal and external validation, it cannot be claimed that this QSAR model is reliable for unknown sample unless its domain of application is defined. If the predictive value of the sample falls into this applicability domain, the value may be considered reliable. The leverage along with the Williams plot is usually used to define applicability domain of a model. The Williams plot defines as the plot of the standardized residuals versus the leverage (*h*). In this plot, two horizontal lines and one vertical line mark a safety area. Compounds with standard residuals>3 standard deviation units and leverage higher than the warning h^*^ are regarded as outliers. The leverage (*h*_i_) of every compound is calculated by following equation:

*h*_i_ *= x*_i_ *(X* ^T^ *X)* ^-1^ *x*_i_^T^

In this equation, *x*_i_ is the descriptor-row vector of the query molecule and* X* is the *k × n* matrix containing the *k *descriptor values for each one of the *n* training molecules. The critical leverage h* (the vertical line) is fixed at *3(k + 1)/n* (24, 25). From the Williams plot (Figure 2), it is obvious that all data points fall within the safety area in model. All compounds have the leverage lower than the warning h^*^ value of 0.62. As a result, it can be said that the model is acceptable for prediction purpose.

*Docking Study*

Docking study was performed on 29 compounds of dataset to explore their interactions with HIV integrase active site and to gain some insight into their binding poses. Docking results indicated that all compounds occupy same space near the co-crystallized ligand (Figure 3). Best docked pose of some selected compounds are shown in Figure 3. As can be seen from Figure 4, compounds bind into the IN active site through two major binding moiety: the carbonyl groups of compounds bind to both Mg^2+^ ions (at distances less than 2.00 Å); aryl side chain groups fit into the protein-DNA interfacial hydrophobic pocket involving π-stacking with the deoxycytosine C16 (DC16) of viral DNA. Figure 5 reveals a high similarity between selected compound 19 and co-crystalized ligand binding modes, suggesting that molecules may show anti-HIV activity via engaging HIV IN active site.

## Discussion

One of the main purposes of QSAR studies is the determination of the factors influencing the activity of the studied compounds. By interpreting the descriptors appeared in the optimization model, it is possible to obtain some insight into the factors that are likely to have effects on the LOG IR. Analyzing the internal and external validation parameters exhibited that SW-MLR model possessed good fitting ability, good predictive ability and high stability.

The relative significance of the descriptors presented in the model was determined based on its standardized regression coefficients. The calculated MLR coefficients cannot be used because the descriptors in final MLR models have not the same units. Standardized regression coefficients of selected descriptors in SW-MLR model are shown graphically in Figure 6. As can be seen, R3u+ is the most significant descriptor with a negative sign, and IDDE has higher coefficient value among descriptors with positive effect on inhibitory activity. An explanation of the selected descriptors using handbook of molecular descriptors follows next (26, 27).

*R3u+*

This descriptor belongs to the GETAWAY R-indices group those are for geometry, topology and atomic-weights assembly. These descriptors are geometrical descriptors in which provide good position of substituents and fragments in molecule (28). In addition, they can carry on good information on molecular size and shape. R3u+ (R maximal auto correlation of lag 3/unweighted) relates to the maximum steric contributions to molecules shape with the topological distance of 3 (29). Since it presented a negative sign in derived linear equation, increasing in value of this descriptor will decrease the activity. 

*R3v+*

R3v+ is defined as R maximal auto correlation of lag 3/weighted by atomic van der Waals Volumes and is derived from the Molecular Influence Matrix (MIM). It contains local or distributed information on molecular structure. In most cases more than one GETAWAY descriptor is needed to reach an acceptable modeling power. The positive coefficient of R3v+ implies that high value of atomic van der Waals volumes can lead to increased activity of a compound (30). 

*IDDE*

IDDE is another topological descriptor that measures the complexity of the molecule in terms of the diversity of elements that includes in its chemical structure, such as the type of atoms, bonds, cycles, etc. The Topological Distance matrix (D), introduced by Harary in the 1960s, accounts for the ‘‘through bond’’ interactions of atoms in molecules; descriptor IDDE characterizes the distribution of the topological distances in each chemical graph (31). The positive coefficient of IDDE suggests that high value of topological distances can lead to increased activity of a compound.

*Mor11m*

Mor11m is a 3D-MoRSE (“Molecule Representation of Structures based on Electron diffraction”) descriptor that is a representation of the three-dimensional structure of a molecule (32). The 3D-MoRSE descriptors were proposed based on electron diffraction studies which are used to prepare theoretical scattering curves (33). The Mor11m has a positive sign, which indicates that the activity value is directly related to this descriptor. Hence, it is concluded that increasing the value of this descriptor causes an increase of anti-HIV activity.

Overall, The generated QSAR model indicated that the inhibitory activities of compounds greatly depend upon molecular geometry, complexity and atomic van der Waals Volumes. For example, quinazoline series displayed higher inhibitory activity in comparison to 2-benzoxazolinone and diazocoumarin derivatives because of molecular gemometry and complexity. In addition, introduction of bulky group such as choro instead of fluoro group contributed to the improvement of the activity in some analogues because of larger atomic van der Waals Volume. 

## Conclusion

QSAR analysis was performed on inhibition rate of HIV p24 expression values of 2-benzoxazolinone, diazocoumarin and quinazoline derivatives by use of the MLR procedure. For each molecule 1444 theoretically derived descriptors were calculated. The best set of calculated descriptors was selected with the stepwise method. The obtained model displayed good statistical power, suggesting significant correlation of molecules with their anti-HIV activities. Based on QSAR model results, molecules geometry, molecules complexity, electron diffraction and van der Waals volumes were found to be important factors controlling the inhibitory activity. Docking analysis also revealed that all compounds fit perfectly in the HIV integrase active site and showed binding modes similar to co-crystallizes ligand. The proposed model and docking data can provide useful vision into some instructions for further designing and synthesizing of new anti-HIV compounds.
